# Long‐term effects of clear‐cutting forestry on ectomycorrhizal fungi in boreal forest

**DOI:** 10.1111/nph.71165

**Published:** 2026-04-03

**Authors:** Björn D. Lindahl, Karina E. Clemmensen, Johan Stendahl, Anders Dahlberg

**Affiliations:** ^1^ Department of Soil and Environment Swedish University of Agricultural Sciences Box 7014 Uppsala SE‐750 07 Sweden; ^2^ Department of Forest Mycology and Plant Pathology Swedish University of Agricultural Sciences Box 7026 Uppsala SE‐750 07 Sweden

**Keywords:** biodiversity, chronosequence, community ecology, forest inventory, soil fungi, succession

## Abstract

- Clear‐cutting is detrimental to ectomycorrhizal fungi in a short time perspective, but long‐term effects on species richness and community composition are uncertain. To evaluate ecological sustainability of rotation forestry, we examined to what extent communities similar to those in old forests develop within the time frame of a rotation period.
- We conducted DNA‐based analyses of ectomycorrhizal fungal communities in > 1500 sites across Swedish boreal forests and used space for time substitution to analyse the development of species richness, community composition, and abundance with increasing stand age, ranging from recent clear‐cuts to 200‐yr‐old forests.
- Ectomycorrhizal fungal species richness peaked in 30‐ to 40‐yr‐old secondary stands, but community composition remained altered in up to 100‐yr‐old stands. Certain species in the Atheliaceae family thrived in secondary forests, whereas many *Cortinarius* and *Russula* species linked to acidic and unfertile soils were more common in old, natural forests.
- While Swedish rotation forestry does not appear to decrease species richness, it will progressively change the composition of ectomycorrhizal fungal communities at the landscape level, with species characteristic of acidic and nutrient‐poor old‐growth boreal forests decreasing in abundance.

## Abstract 2

See also the Commentary on this article by Lõhmus, **251**: 2292–2294.

## Introduction

The composition of ectomycorrhizal fungal communities is shaped by the interplay between persistent conditions of the habitat and abiotic and biotic factors that are more variable over time and space. In this context, human land use has developed into a pervasive and extensive impact, for example by clear‐cut logging in boreal forests. In the prevailing rotation forestry, entire stands are treated uniformly with clear‐felling, soil preparation, planting, clearing, thinning, and with repeated felling expected after 60–100 yr. This is a large change compared with natural boreal forest dynamics (Kuuluvainen & Aakala, 2011). Decreasing populations of many animals, fungi, and plants, because of clear‐cutting forestry, have been reported frequently since the 1980s (Ingelög *et al*., 1987; Berg *et al*., 1994; Sandström, 2015). However, already in 1857, mycologists were concerned about the impact of deforestation on fungal diversity in Sweden (Fries, 1857). Currently, in Fennoscandia, 15% of the *c*. 2000 known ectomycorrhizal fungi are nationally red‐listed, primarily due to declining populations caused by clear‐cutting of forests with long continuity (Nordén *et al*., 2014; Hyvärinen *et al*., 2019; SLU‐Artdatabanken, 2020; Artsdatabanken, 2021). For methodological reasons, red‐listing mainly focusses on less common to rare species and is primarily based on fruitbody observations. Hence, the impact of rotation forestry on the occurrence of more common ectomycorrhizal fungi as mycelium belowground is less investigated and understood. This aspect of forest biodiversity is critical, as the mycelium of the most frequently occurring ectomycorrhizal taxa makes pivotal contributions to decomposition and nutrient cycling (Lindahl *et al*., 2021).

Relative to free‐living saprotrophs, ectomycorrhizal fungi have little capacity to acquire carbon through decomposition of dead organic matter and instead depend on living tree hosts for their survival, that is, they are obligate biotrophs (Lindahl & Tunlid, 2015). It is, therefore, not surprising that clear‐cutting of forests has detrimental short‐term effects on ectomycorrhizal fungal communities (Melin, 1925; Laiho, 1970; Harvey *et al*., 1980; Jones *et al*., 2003). Following forest harvest and in the absence of living trees, ectomycorrhizal fungi perish (Harvey *et al*., 1980; Persson, 1982; Ferrier & Alexander, 1985; Sterkenburg *et al*., 2019). The communities that subsequently establish in the next forest generation consist of a subset of the communities in old forests, with species richness increasing in concert with the ageing of the planted forest (Kranabetter *et al*., 2005; Wallander *et al*., 2010; Kyaschenko *et al*., 2017; Wang *et al*., 2023). However, the long‐term development of ectomycorrhizal fungal species richness and community composition in secondary forest remains uncertain. The time trajectory of community development is important – if the required establishment time for communities that are characteristic of continuity forests is longer than the typical rotation period (i.e. the time for the planted forest to be ready for another harvest), then rotation forestry cannot be considered sustainable with regard to ectomycorrhizal fungal diversity. With long‐lasting impacts of clear‐cutting, maintenance of communities adapted to natural forest dynamics would depend on forest reserves or increased use of other management regimes than rotation forestry (Rosenvald & Lõhmus, 2008; Felton *et al*., 2020). If, on the other hand, communities with similar characteristics as in continuity forests develop before next harvest, ectomycorrhizal species may persist in a rotation forestry landscape through dispersal among stands.

A major proportion (91%; Skogsdata, 2022) of the forest area in Sweden is managed for timber production, and harvesting is almost exclusively by clear‐cutting. The stand age is a central forestry parameter, providing information about the average age of the dominant trees in a specified forest area. In secondary forests, established after clear‐cutting, assessment of stand age is straightforward, as the age structure is uniform. Clear‐cutting forestry commenced already in the 1920s, although only in the 1950s did it become widely adopted as the principal harvest practice (Lundmark *et al*., 2013, 2021). Today, forest stands younger than 80 yr can, thus, be considered as secondary, that is, regenerated (normally planted) after clear‐cutting, and account for 63% of the Swedish forest land (Skogsdata, 2022). Forests with a stand age older than 80 yr, that is, with trees established before the introduction of industrial forestry, are likely to have a long history of continuous tree cover. However, clear‐cutting was also used during the early 20^th^ century (Lundmark *et al*., 2021). Thus, some stands older than 80 yr may also be categorised as secondary forest, and much of the older forest was selectively harvested, resulting in a more uniform age structure; *c*. 90% of Swedish forest can be considered single‐layered (Skogsdata, 2013). For multilayered forests, stand age cannot be directly translated to time since a stand‐replacing disturbance. Nevertheless, inventory plots can be organised as a chronosequence, but the meaning of stand age shifts throughout the sequence.

Species richness of ectomycorrhizal fungi is reduced in young stands established after clear‐cutting or fire, but as root biomass and allocation of photosynthate increases, the enlarging habitat for ectomycorrhizal fungi allows for more species, in line with theories of positive energy–abundance–diversity relationships (Storch *et al*., 2018). Habitat complexity, proposed to promote biodiversity, is also likely to increase as secondary forest age (cf. Loke & Chisholm, 2022). The particular conditions in young stands may select for a narrow subset of species (Jones *et al*., 2003), and priority effects may enable early establishers to persist for long periods of time, hampering establishment of more species‐rich communities (Kyaschenko *et al*., 2017). Although Twieg *et al*. (2007) found as many fungal species on *Pseudotsuga menziesii* root tips in a 26‐yr‐old stand as in older forests, most other studies have found more long‐lasting negative effects of harvesting on ectomycorrhizal fungal species richness. For example, in an inventory of ectomycorrhizal sporocarps in *Tsuga heterophylla* / *Pinus contorta* forests, Kranabetter *et al*. (2005) found that species richness increased with increasing stand age up to 100 yr. Similarly, based on soil metabarcoding, Wallander *et al*. (2010) observed progressively increasing diversity of ectomycorrhizal fungi along a 90 yr *Picea abies* forest chronosequence, Kyaschenko *et al*. (2017) found reduced species richness in up to 50‐yr‐old *Pinus sylvestris* stands compared with older stands, and Wang *et al*. (2023) found reduced diversity of ectomycorrhizal fungi in previously clear‐cut *Pinus koraiensis* forests even 60 yr after harvest relative to > 200‐yr‐old forests. There is, thus, a general picture of increasing richness of ectomycorrhizal fungal species with increasing stand age.

In a review of effects of clear‐cutting on ectomycorrhizal fungi, Jones *et al*. (2003) concluded that there is usually enough inoculum on clear‐cuts to ensure high levels of root colonisation by ectomycorrhizal fungi and shifted focus to the composition of the fungal community that re‐establishes in the secondary forest. Although Bradbury *et al*. (1998) found no clear differences in ectomycorrhizal fungal communities between young (6–19 yr) and relatively undisturbed (90 yr) *P. contorta* stands, most other studies have found clear successional patterns in ectomycorrhizal fungal communities after stand‐replacing disturbances (Visser, 1995; Palfner *et al*., 2005; Twieg *et al*., 2007; Wallander *et al*., 2010; LeDuc *et al*., 2013). Communities often remain altered for many decades after the disturbance. Some overarching trends on the genus level can be discerned, with repeated observations of certain *Suillus*, *Rhizopogon, Leccinum, Tylospora*, *Lactarius*, and *Thelephora* species being particularly abundant in younger stands, and many *Russula, Cortinarius, Tricholoma*, and *Piloderma* species preferring older forests (Kranabetter *et al*., 2005; Varenius *et al*., 2016, 2017; Kyaschenko *et al*., 2017; Spencer *et al*., 2023; Rianhard *et al*., 2025). Jones *et al*. (2003) proposed that species composition should depend as much on community filtering according to the distinct physical, chemical, and biological conditions on clear‐cuts as on changes in sources of fungal inoculation. Soil pH and nitrogen availability increase after clear‐cutting and then decrease in ageing stands (Wallander *et al*., 2010; Gustienė *et al*., 2022). Thus, it seems plausible that species that are more abundant in older stands also are adapted to more acidic and nutrient‐poor soil conditions.

Several studies have described how mycelial production and the relative abundances of ectomycorrhizal taxa in the total fungal DNA pool increases when trees establish and primary production gradually increases after clear‐cutting (Wallander *et al*., 2010; Kyaschenko *et al*., 2017; Choma *et al*., 2021), with a peak already 10–20 yr after tree planting (Wallander *et al*., 2010), and then a decrease in ageing stands (Clemmensen *et al*., 2015; Wang *et al*., 2023). Biomass may, however, be maintained in old forest, if decreased production is compensated by increased mycelial longevity (i.e., decreased biomass turnover; Hagenbo *et al*., 2017).

To address these issues further, we collected soil samples from > 1500 locations distributed systematically across Swedish coniferous forests, in conjunction with the Swedish National Forest and Forest Soil Inventories, and analysed ectomycorrhizal fungal communities by sequencing amplified ITS2 markers. We hypothesised that:

*Richness of ectomycorrhizal fungal species would increase with stand age and generally be higher in continuity forests with older trees (stand age > 80 yr) than in secondary forest (stand age < 80 yr)*.

*Ectomycorrhizal fungal communities would shift with stand age, with* Suillus, Rhizopogon, Leccinum, Tylospora, Lactarius, *and* Thelephora *species being particularly abundant in younger stands, and* Russula, Cortinarius, *and* Piloderma *species occurring more frequently in older stands*.

*Species that are more abundant in older stands would be adapted to more acidic and nutrient poor soil conditions*.

*The relative abundance of ectomycorrhizal species in the total fungal DNA pool would increase rapidly from low values, reaching a peak in early successional stands, and then decline*.

We also aimed to acquire a better picture of the time course of successional development of ectomycorrhizal fungal communities in Swedish coniferous forests, and to establish the time it takes for secondary forests to develop communities that are similar to those of continuity forests.

## Materials and Methods

### Sampling and descriptive data

Sampling was carried out in June–September 2014–2021 in connection with the Swedish Forest Soil Inventory (Fridman *et al*., 2014). The soil inventory collects data from locations that are distributed systematically in a grid across Sweden with a somewhat higher density in the southern parts (mainly motivated by the more variable forest landscape). Our sampling campaign covered a 240 000‐km^2^ area, spanning the entire latitudinal range of the boreal and hemiboreal forest biomes (Supporting Information Fig. S1). We focussed on 1576 sites with coniferous trees accounting for > 75% of the tree basal area. Sampling was restricted to sites with organic topsoil (mor layer; O horizon) that was distinct from the mineral soil (or rock) beneath. Thus, sites with a high contribution of broadleaved trees and/or lacking a mor layer (i.e. mull soils or sites with *Sphagnum* peat) were not included. Data from a subset of the samples were used in previous publications targeting questions about correlations between fungal communities and organic matter stocks (359 sites; Lindahl *et al*., 2021) or niches of a narrow set of ectomycorrhizal decomposer species with respect to forest age and fertility (577 sites; Packard *et al*., 2025). Here, we address the broader question of patterns of ectomycorrhizal fungal diversity.

Data on tree species composition and stand age were retrieved from the Swedish National Forest Inventory (Fridman *et al*., 2014) and were based on measurements within circular plots with a 20 m radius (0.126 ha). Swedish coniferous forest is strongly dominated by two tree species: *Pinus sylvestris* L. and *Picea abies* (L.) H. Karst., and the landscape is dominated by planted monocultures, with mixed species forests being relatively rare. Thus, relative abundances of *Picea* and *Pinus* were bimodally distributed and negatively correlated. At some sites, the conifers were complemented by a low proportion of broadleaved trees (0–25% of basal area), mainly *Betula* species. For recent clear‐cuts, the tree species composition before harvest was used.

Stand age was determined as the basal area‐weighted average age of all trees, implying that in multilayered forests, the largest trees had a disproportional impact on the assigned stand age, so that the estimated stand age was higher than the arithmetic average of all trees (Skogsdata, 2013). Tree ages were estimated visually and verified by increment boring of selected trees. The annual temperature sum, calculated as a function of latitude and altitude (Odin *et al*., 1983), was used as a climate index. This parameter had a bimodal distribution, with high values in southern lowlands and low values in northern uplands, while intermediate values were less common.

Data on soil pH and nitrogen content were collected from the Swedish Forest Soil Inventory. Specifically, samples of the organic topsoil were collected with a 10‐cm‐diameter corer at 1–9 locations along the perimeter of a 60‐cm‐radius circle within the 20‐m‐radius area. After removal of freshly fallen, structurally intact plant litter, cores spanning the entire organic topsoil down to the mineral soil transition (or to rock in sites without mineral soil), were collected until at least 1.5 l of organic matter was obtained (typically pooling material from 3 cores). Carbon and nitrogen concentrations were determined using an elemental analyser (TruMacCN, LECO, St. Joseph, MI, USA), and the nitrogen concentration of organic matter was expressed relative to the carbon content (i.e. as the N : C ratio). The pH in the topsoil was determined in a slurry consisting of 2 g soil and 25 ml deionised water, using an 855 Robotic Titrosampler with an Aquatrode Plus combined pH electrode (Metrohm, Herisau, Switzerland). Separate samples of the organic topsoil were collected for analysis of the fungal community. For this purpose, material from the uppermost 10 cm (excluding the litter layer) was collected and pooled from five locations within a 1‐m‐radius circle (3 m^2^) concentric with the 60‐cm‐radius circle. In total, 50 ml of organic material, including fine roots, was collected from each site. Samples were, on average, frozen within 6.4 d (range 1–47 d) after sampling, freeze‐dried and finely ground in a ball mill. Collection of tree data, soil samples for pH and N analyses, and soil samples for DNA extraction was coordinated and carried out by the same sampling teams.

**Fig. 1:**
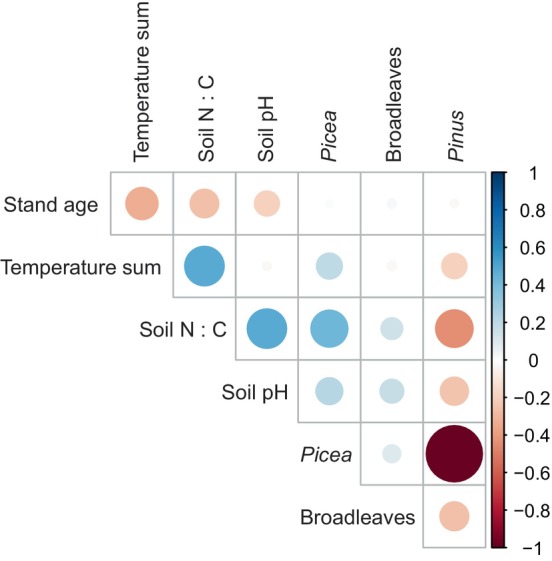
Correlations between parameters of 1576 conifer‐dominated forest sites distributed across Sweden. Blue and red colours indicate positive and negative correlations, respectively, with the size of symbols proportional to the strength of correlation.

Temperature sum, soil N : C, and the proportion of *Picea* correlated positively with each other, forming a gradient in ecosystem fertility with higher abundance of *Picea* on nitrogen‐rich soils towards the south and higher abundance of *Pinus* on nitrogen‐poor soils towards the north. Soil pH and broadleaved trees correlated positively with N : C and *Picea* but were not significantly related to temperature sum. Stand age was negatively correlated with soil N : C, soil pH, and temperature sum but not clearly related to tree species composition (Fig. 1).

### Analysis of fungal communities

DNA was extracted from 50 mg of organic soil, using the NucleoSpin Soil kit (Macherey‐Nagel, Düren, Germany), and the ITS2 region was amplified using the fungal‐specific primers gITS7 and a 3 : 1 mix of the reverse primers ITS4 and ITS4arch, with both forward and reverse primers fitted with unique 8‐bp sample identification tags (Ihrmark *et al*., 2012; Clemmensen *et al*., 2023). DNA extracts were diluted to 0.25 ng μl^−1^ in a 50‐μl PCR volume and amplified using DreamTaq polymerase (Thermo Fisher Scientific, Waltham, MA, USA) with the following cycling programme: 5 min at 95°C; 20–35 cycles of 30 s at 95°C, 30 s at 56°C and 30 s at 72°C; 7 min at 72°C. The number of PCR cycles was optimised for each sample by rerunning samples with too strong or too weak PCR products (according to gel electrophoresis) with cycle numbers adapted to obtain ‘weak but visible’ bands on the gel, as recommended in Castaño *et al*. (2020). Extraction blanks were included in all PCR rounds, and a mock community (Castaño *et al*., 2020) was included in all sequencing libraries, to monitor potential contaminations and sequencing biases. PCR products were run in duplicates, which were pooled and cleaned using the AMPure kit (Beckman Coulter, Indianapolis, IN, USA). DNA concentrations were established using a Qubit fluorometer (Life Technologies, Carlsbad, CA, USA), and DNA from each sample was pooled and purified again using the E.Z.N.A. Omega cycle pure kit (Omega Bio‐Tek, Norcross, GA, USA). Amplicon size distribution was checked using the 2100 Bioanalyzer system (Agilent, Santa Clara, CA, USA), and composite samples were sequenced on the RSII (samples from 2014 to 2016) or Sequel I (samples from 2017 to 2021) platforms (Pacific Biosciences, Menlo Park, CA, USA) by SciLifeLab NGI (Uppsala, Sweden; 2014–2020) and Radboud UMC (Nijmegen, the Netherlands; 2021) after the addition of sequencing adaptors by ligation. The PacBio platform was chosen to minimise bias caused by size variation in the amplicon pool, which is considerable for the fungal ITS2 region (Castaño *et al*., 2020).

Sequences were filtered and clustered using the SCATA pipeline (scata.mykopat.slu.se; Ihrmark *et al*., 2012). Sequences were quality checked to remove those shorter than 100 bp, with mean quality scores lower than 20, with individual bases with a quality score lower than 3, or with a missing 3′ or 5′ tag. Sequences were screened for the gITS7 and ITS4 primers, requiring a minimum match of 90%, and reverse complemented if necessary. Globally unique genotypes were removed to reduce the incidence of sequencing errors. Quality filtering removed 49% of the total sequences, and another 15% were removed as unique genotypes. Remaining sequences were clustered into species hypotheses (hereafter species; Kõljalg *et al*., 2013) through pairwise comparisons using usearch (Edgar, 2010) followed by single linkage clustering, with the minimum similarity to the closest neighbour required to enter a cluster set at 98.75%. This clustering threshold was found to be an optimal trade‐off between separation and merging of closely related sister species among ectomycorrhizal taxa, based on inclusion of representative sequences from the UNITE data base (Abarenkov *et al*., 2024) in the clustering process (passively without affecting clustering). To reduce the impact of long deletions, which sometimes is a component of intraspecific variation, gap elongation penalty was reduced (1, 1, and 0.1 in penalty for substitutions, gap opening and gap elongation, respectively), and homopolymers were collapsed to 3 bp during clustering. Nonfungal sequence clusters were identified and removed by blast comparisons with the NCBI nt database. Ectomycorrhizal fungal species were further identified by blast comparisons of the most frequent genotype with the UNITE database (Abarenkov *et al*., 2024). Sequence clusters were assigned to species when the most frequent genotype had > 99% match with a UNITE sequence in a 0.5% species hypothesis that included a UNITE reference sequence (manually selected by taxonomical expertise), otherwise to genus or family. Details about the identification procedure are supplemented (Notes S1).

### Statistics

The proportion of ectomycorrhizal fungi was calculated as relative abundance among total fungal sequences. Soil pH was log‐transformed (i.e. H^+^ concentrations were double‐log‐transformed) to reduce right‐skewed distribution. Temperature sum, pH, and soil N : C were normalised (subtracting the average and dividing with the SD). Species richness (alpha diversity) of ectomycorrhizal fungi was adjusted for variation in sequencing depth by regressing observed species richness onto the square root of the total number of fungal sequences and then using model residuals as adjusted richness. To avoid negative values, predicted richness (14.4) at the average sequencing depth (1318 reads per site) was added to all values. The total number of detected species at the national level (gamma diversity) was recorded within 15 equally sized (*n* = 105 sites) age classes, and variation in community composition between sites (beta diversity) was calculated for each age class by dividing gamma diversity by average alpha diversity.

Variation in the proportion of ectomycorrhizal fungal sequences among total fungal sequences (square‐root‐transformed to meet the assumption of normally distributed residuals) and in adjusted species richness was analysed with segmented regression (Fong *et al*., 2017), whereby the explaining variable, stand age, was partitioned into two intervals with distinct linear regression coefficients. To account for that old forests are more abundant in the colder regions towards the northeast (Figs 1, S1), temperature sum was included as an additional (nonsegmented) predicting variable. The proportion of *Picea* and the proportion of broadleaved trees were also included as predictors (*Pinus* was not included, due to its strong, negative correlation with *Picea*). Since soil pH and N : C decrease during stand development (Figs 1, S2), and thus partly mask the age effect, models were run both with and without these parameters. Sampling year (2014–2021; categorical) was included to account for the shift in sequencing platform and other interannual variation. The number of days before samples were frozen was also included in the models.

Relative species abundances were calculated among ectomycorrhizal fungal sequences and square‐root‐transformed (Hellinger transformation). For the latter purpose, 53 sites with < 10 ectomycorrhizal fungal sequences were omitted (*n* = 1523). Ordination analyses of ectomycorrhizal fungal community composition were conducted on 166 species detected in 20 sites or more using Canoco 5 (Šmilauer & Lepš, 2014). Species composition of the ectomycorrhizal fungal community was illustrated by correspondence analysis (CA). Sampling year was included as a categorical covariate. Correlation between ectomycorrhizal fungal community composition and stand age was evaluated with canonical CA (CCA), with temperature sum, *Picea*, broadleaves, soil pH, soil N : C, and time to freezing as additional predictors, again with sampling year as a covariate, and tested by 9999 Monte Carlo permutations. To assess the dynamics of changes in ectomycorrhizal fungal community composition with stand age, we ran another canonical correspondence analysis with stand age as the single predictor and then used the sample scores (CaseR scores) on the first and only canonical axis as a plot‐level index of fungal community adaptation to stand age. Temperature sum was included as a covariate, together with sampling year. To estimate the time required for secondary forests to develop ectomycorrhizal fungal communities similar to those of old forests, the ectomycorrhizal community age index was regressed against stand age with an upper hinge segmented model (Fong *et al*., 2017). This model assumed that the index increases linearly with stand age until an estimated breaking point, after which it is constant. Species scores from the same ordination were used to grade species along the age gradient.

Canonical correspondence analyses were also conducted with soil N : C or pH as single predictors. To compensate for generally decreasing soil nitrogen and pH towards the north, temperature sum was included as a covariate, together with sampling year. Species scores from these ordinations were used to assign species niches along nitrogen and pH gradients. To test the hypothesis that ectomycorrhizal fungal species with preference for old forests also tend to occur more commonly on unfertile soil (i.e. with low N : C and pH), correlations between species scores on the stand age and nitrogen or pH axes were tested by linear regression across the 166 included species. To account for the possibility that clear‐cutting has been more common on more fertile sites, the process was repeated with stand age included as a covariate in the CCAs constrained by N and pH.

To investigate the niches of individual ectomycorrhizal fungal species in relation to stand age, the data were subsampled to obtain a balanced number of sites within age classes. We assumed that successional changes are faster at early stages of community development, so stand age was square‐root‐transformed, and the data were split into nine age classes with equal spans in transformed age, after which 67 sites were selected randomly from each of the seven younger classes (*n* = 469). The two oldest classes, which together contained 25 sites older than 200 yr, were removed. The age distributions of sites in which a species was recorded as present were illustrated by violin plots for the 58 species that occurred in > 5% (> 23) of the sites. Species distributions in the subsampled dataset were evaluated using logistic regression models with square‐root‐transformed stand age, untransformed stand age, temperature sum, and tree species (*Picea* relative abundance) as explanatory variables. The best model for each species was chosen by stepwise backward selection based on the Akaike information criterion. Soil N : C and pH were not included as potential predictors of species distributions, since they change during stand development and would interfere with prediction of relations to stand age. Broadleaved trees were also excluded, due to their low contribution to variation.

## Results

Sequencing depth was variable among samples, ranging from 25 to 7950 (average 1318) high‐quality ITS2 reads per site (with global singletons excluded), but 98% of the sites were represented by > 300 reads. Ectomycorrhizal sequences accounted for 0–86% (average 20%) of the fungal sequences and were distributed on 714 species (Table S1).

**Fig. 2:**
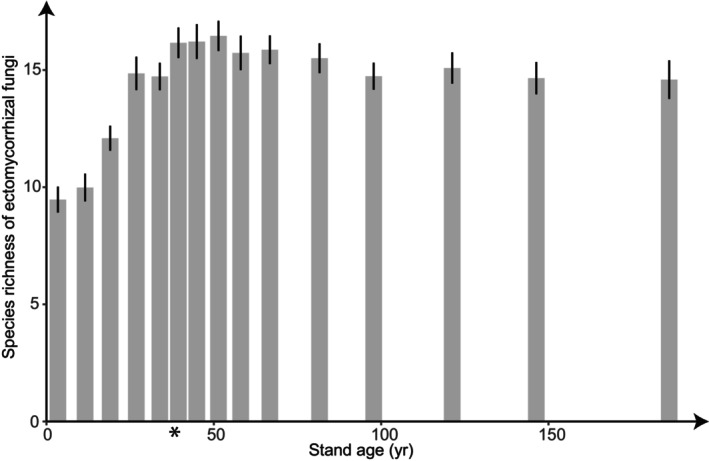
Average species richness (alpha diversity) of ectomycorrhizal fungi in samples of the organic horizon from conifer‐dominated forest sites distributed across Sweden (normalised to a sequencing depth of 1300 reads per sample). The sites were assigned to age classes with equal numbers of sites (*n* = 105) in each class. Error bars denote SE of the mean. *denotes the breaking point of a segmented regression (38 yr), beyond which no further increase in species richness was predicted.

A breaking point in the correlation between species richness of ectomycorrhizal fungi and stand age was estimated at 38 yr (confidence interval: 32–43 yr; Table S2). Before the breaking point, species richness increased with stand age, and after the breaking point, there was a slow but significant negative effect of stand age on richness (Fig. 2). However, in the full model, much of the negative effect of stand age after the breaking point was related to pH, which correlated positively with species richness (Fig. S3) and declined in ageing stands (Figs 1, S2), and the conditional influence of stand age was not significant (*P* = 0.11 in the full model). Tree species had a strong influence on ectomycorrhizal fungal richness in all models. Richness was predicted to be 40% higher in pure *Picea* stands than in pure *Pinus* stands and increased further in the presence of broadleaved trees (Table S2). Temperature sum, N : C, and the number of days it took before samples were frozen were not significant predictors in any of the models of ectomycorrhizal fungal species richness, but sampling year had a significant effect. Gamma diversity (richness per age class) followed a similar pattern as alpha diversity (richness per site), and beta diversity was, thus, quite even across age classes (Fig. S4).

**Fig. 3:**
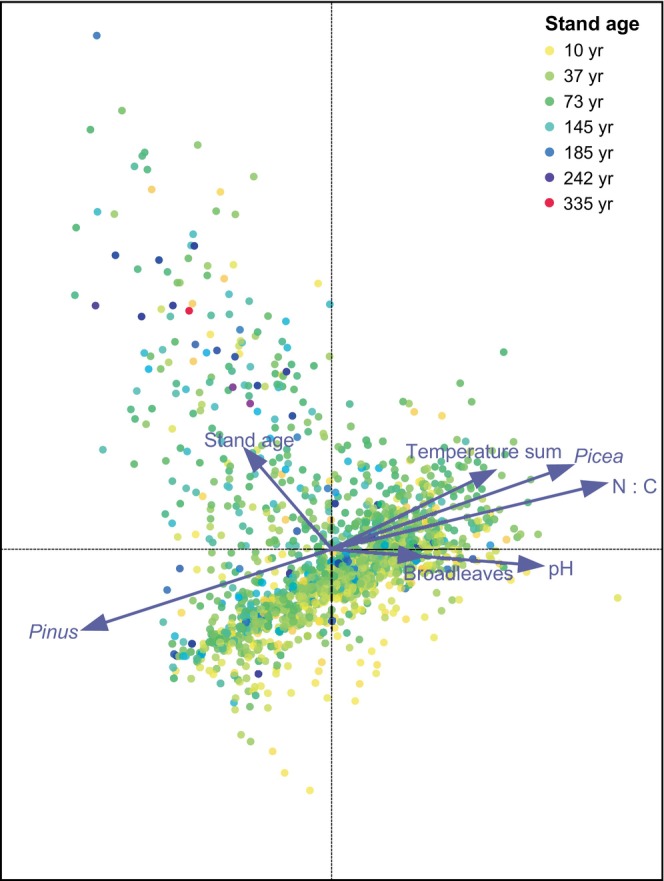
Correspondence analysis of the species composition of ectomycorrhizal fungal communities in the organic horizon of 1523 coniferous forest sites distributed across Sweden. The analysis is based on 166 species that occurred in at least 20 sites. Site symbols are coloured according to the stand age. Site parameter vectors were included as supplementary data, that is they were mapped passively on the ordination.

The species composition of ectomycorrhizal fungi was significantly correlated with soil N : C, soil pH, the relative abundance of *Picea*, temperature sum, stand age, and broadleaved trees in order of declining explanatory power. Neither the order of the predictors, nor their significance, were altered by assessing conditional effects in forward selection, but explanatory power was low – altogether 4.0% (Table S2). Community composition was not significantly related to the time until samples were frozen. Variation in community composition, as illustrated by a correspondence analysis, highlighted a coordinated soil fertility gradient in community variation, in which fungal communities in *Pinus*‐dominated, northern/upland sites (low temperature sum) with less fertile soils (low N : C and pH) diverged from communities in *Picea*‐dominated, southern/lowland forest stands with more fertile soils. Community variation related to stand age was largely orthogonal to this fertility gradient (Fig. 3).

**Fig. 4:**
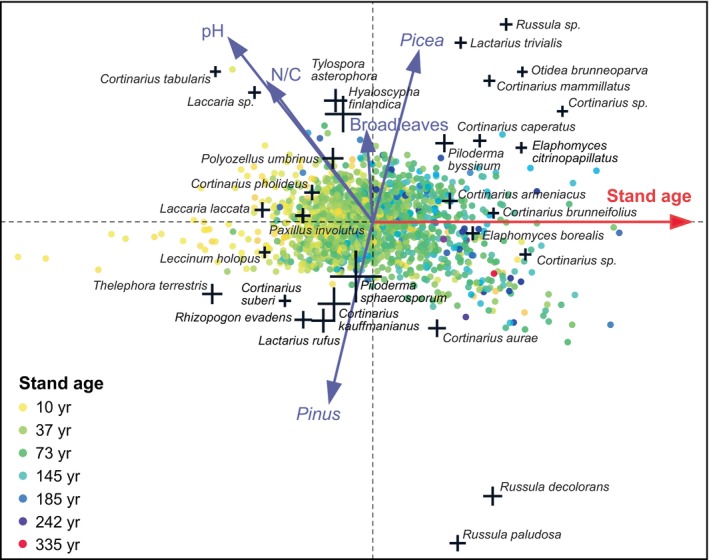
Canonical correspondence analysis of the species composition of ectomycorrhizal fungal communities in the organic horizon of 1523 coniferous forests distributed across Sweden. The analysis is based on 166 species that occurred in at least 20 sites. Stand age was included as the only canonical variable and, thus, aligned with the first ordination axis. Temperature sum was included as a covariate to compensate for the dependency of stand age on geography. The other site parameter vectors were included as supplementary variables, that is, they were mapped passively on the ordination. Positions of site symbols (circles) are derived from the scores of included species, and colours reflect the stand age. The size of species symbols (crosses) reflects frequency of occurrence. For clarity, only the 30 species with highest fit to the canonical ordination axis are included in the figure.

By forcing the first ordination axis to be aligned with stand age in a canonical correspondence analysis, sample scores on the horizontal canonical axis could be used as an ‘ectomycorrhizal community age index’ (Fig. 4). Species scores on the canonical axis indicate which fungal species drove the community age index. A high index value depended particularly on certain *Russula*, *Cortinarius*, and *Elaphomyces* species, while *Thelephora terrestris* and *Laccaria laccata* were important drivers of a low age index.

**Fig. 5:**
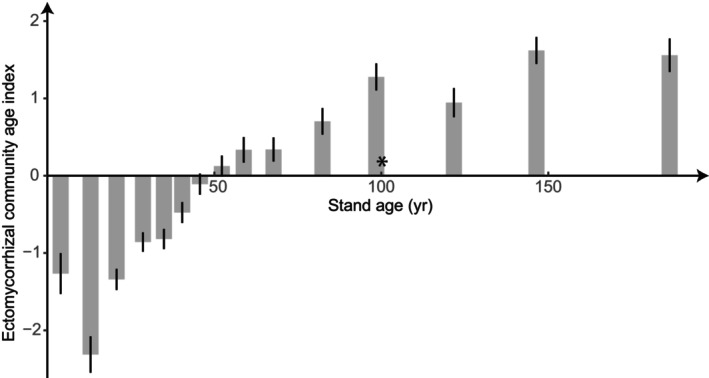
Variation in an ectomycorrhizal community age index across conifer‐dominated forest sites distributed across Sweden. The age index corresponds to the sample scores of a canonical correspondence analysis ordination axis (Fig. 4) and reflects the community composition of ectomycorrhizal fungi in the organic horizon, with positive values indicating high relative abundance of species associated with older stands, and negative values indicating high relative abundance of species associated with young stands. The sites were assigned to age classes with equal numbers of sites (*n* = 101–102) in each class. Error bars denote SE of the mean. *denotes the breaking point (100 yr) of an upper hinge segmented regression, indicating the age beyond which no further change in species composition is predicted.

Community composition, as described by sample scores on the CCA axis constrained by stand age (the ectomycorrhizal community age index; Fig. 4), changed rapidly after clear‐cutting to a minimum value in young secondary forest (9–17 yr; Fig. 5). The index then increased fast with stand age in young forests, then more slowly. A breaking point, defined as the stand age after which the index did not increase further, was estimated to 100 yr (confidence interval: 82–108 yr; Table S2). Species scores on the canonical tree age axis were negatively correlated with species scores on the canonical pH (*P* < 0.0001; *R* ^2^ = 0.11; Fig. S5) and N : C (*P* = 0.0003, *R* ^2^ = 0.08) axes (from separate CCAs). The stand age vs pH correlation was significant also when stand age was included as a covariate in the pH‐constrained CCA (*P* = 0.005, *R* ^2^ = 0.05).

**Fig. 6:**
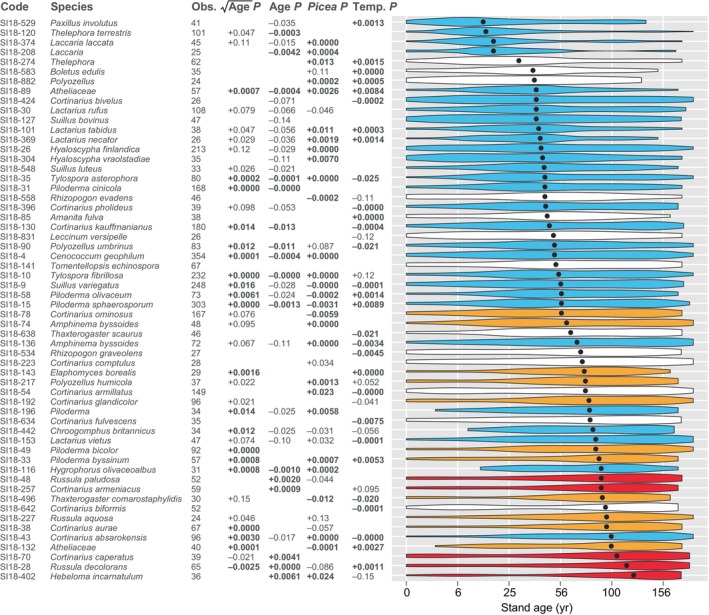
Niches of the 58 most frequent species of ectomycorrhizal fungi in relation to stand age, tree species preference and climate (numbers to the left are study‐specific species IDs). The width of the bars reflects the distribution of observations along a stand age gradient. The plot is based on data that were randomly subsampled to obtain 469 sites evenly distributed along a square‐root‐transformed stand age gradient. The number of observations (Obs.) of each species (*n* out of 469) is given, and median ages of forests with occurrences are indicated by black dots. *P*‐values are given for logistic regression models, in which species occurrences were explained by square‐root‐transformed stand age, untransformed stand age, proportion of *Picea* and temperature sum (Temp.), subjected to backwards predictor selection. The signs of correlations are given before the *P*‐values, and significant relationships (Benjamini–Hochberg‐corrected for multiple tests) are indicated in bold. For species marked in blue, occurrence was negatively correlated with nontransformed stand age. For species marked in orange, occurrence was positively correlated with square‐root‐transformed stand age and not negatively correlated with untransformed stand age. For species marked in red, occurrence was positively correlated with untransformed stand age. Species marked in white were not significantly correlated with stand age. All relations to stand age are conditional on correlations with tree species and climate.

Most of the frequent species increased in occurrence during the early part of the chronosequence, as indicated by a positive correlation with square‐root‐transformed stand age (Fig. 6). Many species, *Thelophora terrestris, Laccaria* spp., *Cortinarius kauffmanianus*, *Polyozellus umbrinus, Cenococcum geophilum, Hygrophorus olivaceoalbus*, and certain species in the *Atheliaceae* family: *Tylospora asterophora, T. fibrillosa, Piloderma cinicola*, and *P. sphaerosporum*, declined in the oldest stands, as indicated by a negative correlation with untransformed stand age, leading to unimodal distributions with highest frequencies in < 60‐yr‐old stands. By contrast, *Elaphomyces borealis, Piloderma bicolor, P. byssinum, Russula paludosa, R. decolorans*, *Cortinarius armeniacus, C. aurae, C. caperatus*, and *Hebeloma incarnatulum* showed a preference for old stands, as indicated by only a positive correlation with stand age (either square‐root‐transformed or nontransformed).

**Fig. 7:**
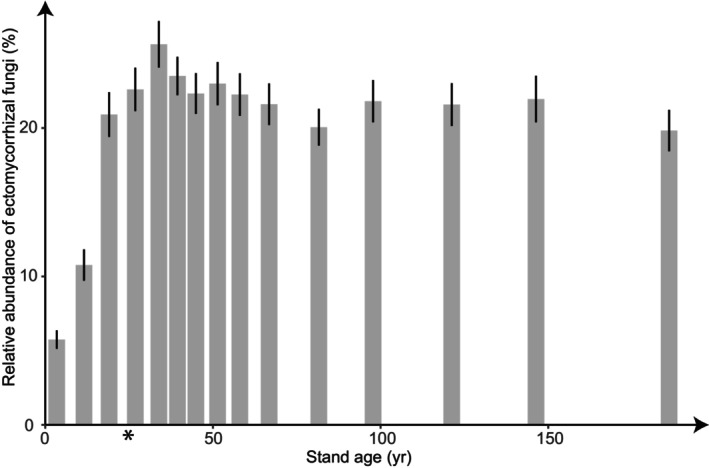
Average relative abundance of fungal ITS markers assigned to ectomycorrhizal species in the organic horizon of conifer‐dominated forest sites distributed across Sweden. The sites were assigned to age classes with equal numbers of sites (*n* = 105) in each class. Error bars denote SE of the mean. *denotes the breaking point of a segmented regression (25 yr).

A breaking point in the correlation between relative abundance of ectomycorrhizal fungi and stand age was estimated at 25 yr (confidence interval: 21–29 yr; Table S2). Before the breaking point, relative abundance increased steeply, and after the breaking point, there was a slow decline with further increasing stand age (Fig. 7). After the breaking point, stand age had a significant negative effect on ectomycorrhizal fungal relative abundance regardless of whether N : C and pH were included as additional predictors. Tree species had a strong influence, with relative abundance of ectomycorrhizal fungal sequences predicted to be 27% in 25‐yr‐old pure *Picea* forests and 17% in pure *Pinus* forests. There was also a significant positive correlation with N : C, and a marginally significant negative correlation with temperature sum, but neither pH nor the number of days it took before samples were frozen were significant predictors. Sampling year had a significant effect on the relative abundance of ectomycorrhizal fungi.

## Discussion

### Species richness

In contrast to our hypothesis (H1) that ectomycorrhizal fungal species richness would be generally reduced in secondary forest (Kranabetter *et al*., 2005; Kyaschenko *et al*., 2017; Wang *et al*., 2023), we found relatively high species richness 3–5 decades after clear‐cutting (Fig. 2). This was also reflected in gamma diversity (Fig. S4), that is, when forest across Sweden were grouped in age classes and species richness was assessed for each class as a whole; ageing secondary stands harboured the highest number of species (although the lack of replication of age classes contributes uncertainty to the analysis). The increase in species richness during development of secondary stands is due to progressive recruitment of new species to the community by dispersal in combination with habitat expansion for ectomycorrhizal fungi as tree age, net primary production, and size of the root systems increase (Storch *et al*., 2018). The species richness of ectomycorrhizal fungi has previously been found to correlate with tree biomass (Luoma *et al*., 2004; Sterkenburg *et al*., 2019). After a peak in species richness, we found a slight but significant decrease in ageing stands (−15% after 200 yr; Fig. 2). With pH included as predictor in a statistical model of ectomycorrhizal fungal species richness, the negative effect of stand age after the breaking point was not significant (Table S2). Species richness was also markedly higher in *Picea*‐dominated forests, which are usually less acidic than *Pinus*‐dominated forests and often contain more broadleaved trees (Fig. 1). However, in contrast to pH, tree species composition could not replace age as a predictor of the decline in species richness in older forests. Further, there was no significant relation between species richness and N : C. This suggests that, as stands develop and pH progressively decreases, the increasingly acidic conditions push fungal communities towards a somewhat lower richness and a higher contribution of more acido‐tolerant species. In a global study, pH was the best predictor of ectomycorrhizal fungal species richness with a positive sign (given a high abundance of suitable hosts; Tedersoo *et al*., 2014). However, pH in ageing stands decreases in concert with decreasing nitrogen availability, declining primary production and increasing competition with ericoid mycorrhizal plants and fungi (Fanin *et al*., 2022), and manipulative experiments are needed to disentangle the relative importance of different drivers.

### Community composition

While species richness in secondary stands reached similar or even higher levels as in continuity forests well within a rotation period, community composition in secondary stands was consistently different from that in old continuity forests, even in up to 100‐yr‐old stands (Fig. 5). These results support our hypothesis (H2) that ectomycorrhizal fungal communities would change systematically with stand age, corroborating earlier studies (Kranabetter *et al*., 2005; Varenius *et al*., 2016, 2017; Kyaschenko *et al*., 2017; Spencer *et al*., 2023), but whereas earlier studies were restricted to a few sites, our large‐scale sampling provided a better resolved picture of community dynamics and indicated that impacts of forestry on ectomycorrhizal fungal community composition are even more long‐lasting than previously suggested. As communities in secondary forests on average are different from those of continuity forest, even when stands reach maturity and are ready for another harvest, our large‐scale, systematic sampling of Swedish forests indicates that rotation forestry progressively shifts fungal community composition at the landscape level, with species that are selected for by clear‐cutting and subsequent forest management becoming more abundant and sensitive species gradually declining.

The observed species patterns largely follow previous findings, with species in the genera *Cortinarius, Russula*, and *Hebeloma* standing out as being more frequent in forests with long continuity (Figs 4, 6). These genera have retained a genetic capacity to produce extracellular peroxidases that may be used to mobilise nitrogen from particularly recalcitrant organic matter (Bödeker *et al*., 2014; Miyauchi *et al*., 2020), and *Cortinarius aurae* has even been linked to accelerated decomposition and low organic matter stocks across Swedish boreal forest (Lindahl *et al*., 2021). Already Last *et al*. (1987) proposed that ectomycorrhizal fungi adapted to later forest successional stages would be better adapted to acquire resources from recalcitrant litter. The high capacity of many of the species that were particularly common in old stands to mine for organic nitrogen corresponds with their tendency to preferentially occur on unfertile soils (low pH and N : C), corroborating our hypothesis (H3). However, the correlation was stronger for pH than for soil nitrogen content, further supporting that progressively decreasing pH during stand development selects for acido‐tolerant species in old forests, but this theory calls for experimental testing. While there was a clear trend of most ectomycorrhizal decomposers to occur more frequently in older stands, some species (notably, *Cortinarius ominosus* and *Cortinarius kauffmanianus*) were also common in younger stands, suggesting that a few species maintain the function of ectomycorrhizal decomposition in secondary forests (Packard *et al*., 2025).

Several species in the Atheliaceae family, *Piloderma cinicola, Piloderma sphaerosporum, Tylospora asterophora*, and *Tylospora fibrillosa*, as well as *Cenococcum geophilum*, thrived in intermediately aged stands. Their distribution patterns were best described by second‐order regressions, with increasing probability of detection as young stands develop, but declining probabilities in old stands, and had median occurrences at *c*. 60 yr (Fig. 6). The proliferation of species within Atheliaceae in managed forests corroborates the findings of Wallander *et al*. (2010) and Kyaschenko *et al*. (2017), but the present study had a hundredfold more samples and a much larger area of investigation. Previously, the genera *Piloderma* and *Tylospora* were also found to have intermediate distributions along a Swedish forest fertility gradient; they were preferably found in more nutrient‐poor forests than, for example *Thelephora* and *Laccaria*, but in more nutrient‐rich and less acidic forest than *Cortinarius* (Sterkenburg *et al*., 2015). There are also some indications that *Piloderma* species, along with *Thelephora, Rhizopogon, and Suillus* species, may form a spore bank (Glassman *et al*., 2015), which could facilitate rapid establishment after clear‐cutting. Once established, these pioneer species may persist for a long time due to priority effects, which are expected to be strong in ectomycorrhizal fungal communities (Kennedy *et al*., 2009).

Some of the species that thrive in recently harvested sites, for example *Thelephora terrestris* and *Laccaria laccata*, almost disappeared along the chronosequence, but the most frequent species, for example *Cenococcum geophilum, Piloderma sphaerosphorum, Tylospora fibrillosa*, and *Hyaloscypha finlandica*, had broad ranges and occurred in both secondary and continuity forests. Furthermore, there is some evidence of niche differentiation within genera. For example, *Cortinarius ominosus* and *Cortinarius kauffmanianus* had a broad range with a median occurrence in relatively young stands, whereas *Piloderma byssinum* and *Piloderma bicolor* were preferably found in old stand, in contrast to other species in their respective genera (Fig. 6). Some of the indicator species of young stands (*Cortinarius tabularis, Cortinarius pholideus, Cortinarius bivelus, Leccinum holopus*; Figs 4, 6) associate with *Betula* species, which are common pioneers after clear‐cutting. *Suillus brevipes* was abundant in young *Pinus banksiana* stands in North America (Visser, 1995; LeDuc *et al*., 2013), but in this study, *Suillus* species occurred evenly across the chronosequence.

### Relative abundance

We hypothesised (H4) that the relative abundance of ectomycorrhizal species in the total fungal DNA pool would increase rapidly from low values, reaching a peak in early successional stands, and then decline (Wallander *et al*., 2010; Kyaschenko *et al*., 2017; Choma *et al*., 2021). Indeed, across Swedish coniferous forests, the average relative abundance of ectomycorrhizal fungal species peaked at just over 20% of the fungal ITS sequences, already 25 yr after forest was re‐established after clear‐cutting, and then decreased significantly in older stands (Fig. 7). We did not have the opportunity to measure fungal biomass in the inventory samples, but previous studies have found ergosterol concentrations in the organic horizon to be quite stable among differently aged stands (Kyaschenko *et al*., 2017), so we assume that the total fungal biomass did not vary systematically across the age gradient, and that the observed pattern in relative sequence abundance roughly reflected variation in ectomycorrhizal mycelial biomass. High mycorrhizal abundance was, thus, reached considerably earlier than the maximum in net primary production, which commonly takes > 50 yr to develop (Gundale *et al*., 2024). Ectomycorrhizal abundance was also positively correlated with soil N : C, and with *Picea* that commonly grow on more nutrient‐rich soils (Table S2; Fig. 1). In Swedish boreal forest, growth of ectomycorrhizal mycelium is often constrained by low‐nitrogen availability (Kalliokoski *et al*., 2010; Högberg *et al*., 2021). Furthermore, in a chronosequence spanning over several thousands of years, the relative abundance of ectomycorrhizal fungi decreased with increasing time since disturbance, whereas root‐associated ascomycetes – mainly ericoid mycorrhizal fungi – increased their dominance and suppressed ectomycorrhizal fungi (Clemmensen *et al*., 2015; Fanin *et al*., 2022).

### Caveats

Our study suffers from problems associated with space‐for‐time substitution. The increase in the ectomycorrhizal community age index at 100 yr (Fig. 5) may partly reflect that previously clear‐cut stands are rare in the older forest classes. Thus, we cannot exclude that effects of clear‐cutting persist for even longer time. At the same time, management disturbances during stand development (e.g. thinning, which was not included in our analyses) may sustain the distinct communities of previously clear‐cut stands in maturing forests.

Furthermore, a longer history of intense forestry in the southern parts of Sweden, which are more densely populated and have more productive forests, led to a negative correlation between temperature sum and stand age (Figs 1, S1), which was accounted for in statistical analyses by including temperature sum as a covariate. For example, the alpine species *Cortinarius absarokensis* was primarily detected in old forests, but its age‐niche was mainly explained indirectly by a preference for cold climate (Fig. 6). Stand age was also negatively correlated with soil fertility (N : C and pH; Fig. S2), and it is possible that this correlation was, partly, the result of selective harvesting leading to younger stands on more fertile soils. However, based on evidence from local time series (Wallander *et al*., 2010), we argue that the opposite causality is likely to be more important, that is that pH and N : C are elevated after clear‐cutting and then gradually decline as stands age. Thus, the tendency for decreasing abundance and species richness of ectomycorrhizal fungi with increasing stand age (after the breaking point) may be partly due to stand selection during management, but our conclusion is that time‐dependent changes in site conditions were likely to be more important. Tree species composition was not strongly correlated with stand age, presumably because it is primarily regulated by management rather than by ecological succession.

Our community data were noisy, but the large number of observations enabled us to draw reliable conclusions based on aggregated data, although the statistical models over‐all had low predictive power. An important reason is that every community sample was based on only five soil cores distributed over *c*. 3 m^2^, and given the high spatial patchiness of ectomycorrhizal fungal communities (Pickles *et al*., 2010), the stochastic component is large. This is also evident in the large variability in the ectomycorrhizal community age index, which often attained low values also in old stands and vice versa (Fig. 3), either due to random sampling of fungal species outside their typical niche or due to divergent local conditions at the sampling spot (e.g. natural disturbances in old forest or retained trees/edge effects on clear‐cuts). To derive a reliable index value for a single stand, pooling of many soil samples from a relatively large area would be required (Mielke, 2022). Furthermore, with our random sampling of soil DNA the probability of detecting rare species is low despite large sample numbers. We detected 443 ectomycorrhizal fungal species with conspicuous sporocarps, whereas the number of known macro‐fungal ectomycorrhizal species linked to coniferous forests in Sweden amounts to at least 720 (SLU Artfakta, 2025). Only 22 of the 146 nationally red‐listed, conifer‐associated ectomycorrhizal fungi in Sweden (SLU Artdatabanken, 2020) were detected.

### Conclusions

The relative abundance of ectomycorrhizal species in the fungal DNA pool increased to similar levels as in old continuity forests within two decades after clear‐cutting. Young stands were characterised by a relatively species poor community, which contained pioneer taxa (e.g. *Thelephora terrestris* and *Laccaria laccata*) that are favoured by the disturbance. In the following decades, species richness increased to a somewhat higher level than in old continuity forest stands, potentially linked to increased pH after harvesting, and certain *Piloderma* and *Tylospora* species proliferated. As soils progressively got more acidic and less fertile with increasing stand age, ectomycorrhizal fungal species richness declined somewhat, selecting for a community characterised by taxa with capacity to exploit recalcitrant organic soil resources – notably *Cortinarius*, *Hebeloma*, and *Russula* species. This means that clear‐cutting forestry is likely to be sustainable with regards to the biomass and species richness of ectomycorrhizal fungi, which are restored well within the time limits of a rotation period and even reach somewhat higher levels than in seminatural forests. However, clear‐cutting has large effects on the community composition of ectomycorrhizal fungi that last for at least a century. Many species that occur frequently in old continuity forests are negatively affected by rotation forestry. This means that rotation forestry progressively changes the ectomycorrhizal fungal community at the landscape level, and this process is likely to accelerate with repeated clear‐cutting (Lunde *et al*., 2025). The new community may be better adapted to the more fertile soil conditions of managed forests (Jones *et al*., 2003), but many of the species that are characteristic of the predominantly nutrient poor and acidic boreal forests decrease in abundance. To what degree this ongoing change alters nutrient cycling processes driven by ectomycorrhizal fungi remains to be investigated. The declining community is likely to contain many rare species, which largely escaped detection by our random sampling and may risk getting locally extinct as transformation of the forest landscape proceeds. If there is a desire to reduce the impact of forestry on ectomycorrhizal fungi, continuous cover forestry with higher levels of tree retention at harvest is an option (Sterkenburg *et al*., 2019; Lariviere *et al*., 2025), but enough trees must be retained to prevent major postharvest increases in pH and soil fertility, and competitive exclusion of ectomycorrhizal fungal species adapted to unfertile soils.

## Competing interests

None declared.

## Author contributions

BDL, JS and AD planned and performed the research. BDL analysed the data and drafted the manuscript. BDL, KEC, JS and AD all participated in the interpretation of results and writing of the paper.

## Disclaimer

The New Phytologist Foundation remains neutral with regard to jurisdictional claims in maps and in any institutional affiliations.

## Supporting information


**Dataset S1** Data on sampling year, temperature sum, soil pH, soil N : C, stand age, share of *Picea*, share of *Pinus*, share of broadleaved trees, days until samples were frozen, and sample coordinates.

**Dataset S2** Counts of sequences assigned to ectomycorrhizal fungal species and samples, as well as the total number of fungal sequences for each sample.

**Fig. S1** Map of Sweden with sampling points marked in colours indicating stand age or temperature sum.
**Fig. S2** Average pH and N : C in samples of the organic horizon from conifer‐dominated forest sites distributed across Sweden.
**Fig. S3** Average species richness (alpha diversity) of ectomycorrhizal fungi in samples of the organic horizon from conifer‐dominated forest sites distributed across Sweden.
**Fig. S4** Gamma diversity and beta diversity of ectomycorrhizal fungi in the organic horizon from conifer‐dominated forest sites distributed across Sweden.
**Fig. S5** Species scores from a CCA analyses with stand age as predictor plotted against species scores from a CCA analyses with pH as predictor.
**Notes S1** Details about taxonomic identification of ITS sequences.

**Table S1** Representative ITS2 sequences, UNITE species hypotheses, and taxonomic identifications of all detected ectomycorrhizal species.

**Table S2** Output of statistical analyses.
Please note: Wiley is not responsible for the content or functionality of any Supporting Information supplied by the authors. Any queries (other than missing material) should be directed to the *New Phytologist* Central Office.

## Data Availability

The raw sequence data that support the findings of this study are openly available in NCBI SRA under the accessions no. PRJNA693127. All other data are available in the Supporting Information of this article (Datasets S1, S2).
