# Supplementary material for: Long‐term effects of clear‐cutting forestry on ectomycorrhizal fungi in boreal forest
Source: New Phytol. 2026 Apr 3;251(5):2818–31. doi: 10.1111/nph.71165 (PMC13443205; doi:10.1111/nph.71165)
Supplement: Supplementary file 1 [file NPH-251-2818-s003.docx]

## *New Phytologist* Supporting Information

Article title: Long-term effects of clear-cutting forestry on ectomycorrhizal fungi in boreal forest

Authors: Björn D. Lindahl, Karina E. Clemmensen, Johan Stendahl, Anders Dahlberg

Article acceptance date: 13 March 2026

The following Supporting Information is available for this article:

**Fig. S1** Map of Sweden with sampling points marked in colours indicating **a)** stand age or **b)** temperature sum.

**Fig. S2** Average **a)** pH and **b)** N/C in samples of the organic horizon from conifer-dominated forest sites distributed across Sweden. The sites were assigned to age classes with equal numbers of sites (n=105) in each class. Error bars denote standard error of the mean.

**Fig. S3** Average species richness (alpha diversity) of ectomycorrhizal fungi in samples of the organic horizon from conifer-dominated forest sites distributed across Sweden (normalized to a sequencing depth of 1300 reads per sample). The sites were assigned to pH classes with equal numbers of sites (n=105) in each class. Error bars denote standard error of the mean.

**Fig. S4** **a)** Gamma diversity and **b)** beta diversity of ectomycorrhizal fungi in the organic horizon from conifer-dominated forest sites distributed across Sweden. Sampled sites were assigned to are classes with equal numbers of sites (n=105) in each class.

**Fig. S5** Species scores from a CCA analyses with stand age as predictor (y-axis) plotted against species scores from a CCA analyses with pH as predictor. The plot displays data from 166 species that occurred in at least 20 sites. The line represents a linear regression (R^2^ = 0.11; *P* < 0.0001).

**Table S1** Representative ITS2 sequences, UNITE species hypotheses, and taxonomic identifications of all detected ectomycorrhizal species.

**Table S2** Output of statistical analyses.

**Note S1** Details about taxonomic identification of fungal ITS sequences.

In total, 3829162 sequences were assembled into 15249 non-singleton clusters. Of these, 3442 clusters that accounted for at least 1% of the sequences in at least one site were identified manually and accounted for 96.2% of the sequences. Initially, they were identified at the kingdom level by BLAST comparison of the most frequent genotype in each cluster against the NCGI nt database. Among the identified clusters, 3139 fungal clusters contained 3017630 sequences (78.8% of total) and 303 non-fungal clusters contained 667391 sequences (17.4% of total). The remaining 144141 unidentified sequences (3.8% of total) were considered fungal, although they included a minor proportion of non-identified non-fungal sequences. However, assuming that the proportion of non-fungal sequences among the non-identified clusters was the same as among the identified clusters, the contribution of non-fungal sequences to total fungal sequence pool was estimated to only 0.7%.

The 3139 fungal clusters were further identified manually, based on a combination of BLAST searches of representative sequences against the UNITE database and neighbour joining trees, produced in MEGA X (Kumar et al. 2018). Clusters were assigned to species when the most frequent genotype had >99% match with a sequence in a 0.5% species hypothesis in UNITE that was annotated by a taxonomic expert. Clusters that could not be identified reliably to species were assigned to genera or families, based on their placement in bootstrapped neighbour joining trees with identified reference sequences.

Identified species were categorised as ectomycorrhizal based on prevailing views on ecological strategies established at the genus level (Põlme et al. 2021). For identified species in ambiguous genera/families containing both ectomycorrhizal species and other lifestyles, we consulted taxonomic expertise through personal communication (see below). Among the identified fungal clusters, 611 (19%) were classified as ectomycorrhizal.

Lowly abundant ectomycorrhizal clusters (those that did not account for >1% of sequences in any of the sites) were identified by local BLAST searches of representative sequences from the 13809 non-identified clusters against representative sequences from the 3139 identified fungal clusters. Clusters with best matches to sequences identified as ectomycorrhizal were further analysed manually, as described above. This process identified 607 additional ectomycorrhizal clusters, summing up to a total of 1218. Of these, 714 were detected in the samples used in this study.

The following groups were more closely investigated:

Albatrellaceae: Three clusters with affinity to the ectomycorrhizal genus *Byssoporia* were considered mycorrhizal.

Atheliaceae: 11 clusters in the ectomycorrhizal *Amphinema*/*Tylospora* clade were identified at the family level, since the phylotaxonomy of the clade is not resolved. A cluster with affinity to the ectomycorrhizal genus *Tretomyces* matched with a UNITE species hypothesis with many sequences obtained from ectomycorrhizal root tips.

Elaphomycetaceae: A cluster with some affinity to the ectomycorrhizal *Elaphomyces* genus matched with a UNITE species hypothesis with sequences obtained from ectomycorrhizal root tips.

*Entoloma*; Four clusters, including the species *Entoloma sericatum* and *Entoloma melenosmum,* formed a well-supported clade and were considered ectomycorrhizal (otherwise the genus *Entoloma* mainly contains saprotrophic species).

*Hyaloscypha*: Among the clusters that could be identified to species, *Hyaloscypha finlandica* and *Hyaloscypha vraolstadiae* are well-established as ectomycorrhizal. Although there is some evidence that *Hyaloscypha variabilis* can from ectomycorrhiza, the species is primarily forming ericoid mycorrhiza, and we did not categorise it as ectomycorrhizal. 10 *Hyaloscypha* clusters that could not be identified to species were considered ectomycorrhizal, since they matched with UNITE species hypotheses with many sequences obtained from ectomycorrhizal root tips (and no or few sequences from roots of ericoid mycorrhizal plants).

Hydnaceae: 28 clusters, including *Clavulina coralloides, Hydnum melitosarx, Sistotrema albopallescens, Sistotrema muscicola*, formed a well-supported clade and were considered ectomycorrhizal (otherwise, the unresolved genus *Sistotrema* mainly contains saprotrophic species).

*Sebacinales*: 13 clusters, including *Sebacina dimitica* and a *Helvellosebacina* sp., formed a well-supported clade within *Sebacina*, and 9 clusters formed a well-supported clade within *Serendipita*. These were considered ectomycorrhizal, since they matched with UNITE species hypotheses with many sequences obtained from ectomycorrhizal root tips (and no or few sequences from ericoid mycorrhizas, orchid mycorrhizas or from roots of herbs/grasses).

References:

Kumar S, Stecher G, Li M, Knyaz C, Tamura K. 2018. MEGA X: Molecular Evolutionary Genetics Analysis across Computing Platforms. *Molecular Biology and Evolution* **35**: 1547–1549.

Põlme S, Abarenkov K, Nilsson RH, Lindahl BD, Clemmensen KE, Kauserud H, Nguyen N, Kjoller R, Bates ST, Baldrian P *et al.* 2021. FungalTraits: a user-friendly traits database of fungi and fungus-like stramenopiles. *Fungal Diversity* **105**: 1–16.

**Dataset S1** Data on sampling year, temperature sum, soil pH, soil N/C, stand age, share of *Picea*, share of *Pinus,* share of broadleaved trees, days until samples were frozen, and sample coordinates (the exact locations of the National Forest Inventory plots are secret, and coordinates are shifted randomly by up to 1000 m).

**Dataset S2** Counts of sequences assigned to ectomycorrhizal fungal species and samples, as well as the total number of fungal sequences for each sample.
